# Knowledge, Perceived Beliefs, and Preventive Behaviors Related to COVID-19 Among Chinese Older Adults: Cross-Sectional Web-Based Survey

**DOI:** 10.2196/23729

**Published:** 2020-12-31

**Authors:** Ying Chen, Rui Zhou, Boyan Chen, Hao Chen, Ying Li, Zhi Chen, Haihong Zhu, Hongmei Wang

**Affiliations:** 1 Department of Social Medicine of School of Public Health, and Department of Pharmacy of the First Affiliated Hospital Zhejiang University School of Medicine Hangzhou, Zhejiang Province China; 2 Department of Public Health Xi’an Medical University Xi’an, Shaanxi Province China; 3 State Key Laboratory for Diagnosis and Treatment of Infectious Diseases National Clinical Research Center for Infectious Diseases, and Collaborative Innovation Center for Diagnosis and Treatment of Infectious Diseases, The First Affiliated Hospital Zhejiang University School of Medicine Hangzhou, Zhejiang Province China

**Keywords:** COVID-19, knowledge, perceived beliefs, behaviors, elderly, China

## Abstract

**Background:**

The COVID-19 pandemic continues to pose an international public health threat. Prevention is of paramount importance to protect the high-risk group of older adults until specific treatments for COVID-19 become available; however, little work has been done to explore factors that promote preventive behaviors among this population.

**Objective:**

This study aims to investigate the knowledge, perceived beliefs, and preventive behaviors towards COVID-19 of older adults in China and determine the factors that influence their practice of preventive behaviors.

**Methods:**

From February 19 to March 19, 2020, a cross-sectional, web-based survey was administered to Chinese older adults in all 31 provinces of mainland China using a convenience sampling method to assess the respondents’ knowledge, perceived beliefs, and preventive behaviors towards COVID-19. Standard descriptive statistics and hierarchical linear regression analyses were conducted to analyze the data.

**Results:**

A total of 1501 participants responded to the survey, and 1263 valid responses (84.1%) were obtained for further analysis. The overall correct rate on the knowledge questionnaire was 87%, overall positive beliefs regarding COVID-19 were found, and the mean behavior score was 13.73/15 (SD 1.62, range 5-15). The hierarchical linear regression showed that respondents who were married or cohabitating and who lived in areas with community-level control measures were more likely to practice preventive behaviors (*P*<.01). Knowledge (*β*=0.198, *P*<.001), perceived susceptibility (*β*=0.263, *P*=.03), perceived benefits (*β*=0.643, *P*<.001), and self-efficacy in preventing COVID-19 (*β*=0.468, *P*<.001) were also found to be significantly associated with preventive behaviors.

**Conclusions:**

Most older residents had adequate knowledge and positive beliefs regarding COVID-19 and engaged in proactive behaviors to prevent the disease. Knowledge and beliefs were confirmed to be significantly associated with behavior responses. Our findings have significant implications in enhancing the effectiveness of COVID-19 prevention programs targeting the older population; these programs must be continued and strengthened as the epidemic continues.

## Introduction

COVID-19, which was first detected in Wuhan, China, in December 2019, is an emerging infectious disease caused by a novel coronavirus (SARS-CoV-2). The crude case fatality rate of COVID-19 was 3.7% worldwide as of August 9, 2020 [1], which is much lower than that of severe acute respiratory syndrome (SARS, 9.5%), Middle East respiratory syndrome (MERS, 34.4%), or H7N9 (39.0%) [2]; however, evidence has shown that SARS-CoV-2 is more contagious than the viruses that cause the latter diseases [3,4]. Considering its extensive spread scope, COVID-19 was declared a public health emergency of international concern on January 30, 2020 [5] and was declared a pandemic on March 11, 2020 [6] by the World Health Organization (WHO). As of August 9, 2020, the virus had reached 215 countries and regions, resulting in over 19 million confirmed infections and more than 700,000 deaths [1].

Older people are especially susceptible to infectious diseases due to decreased immunity [7], the presence of underlying chronic illnesses [8], and cognitive impairment, which causes difficulty in their participating in proper prevention activities [9]. In the case of COVID-19, older people are also at significantly higher risk of morbidity and mortality [10-12]. According to an epidemiological study on COVID-19 in China, people aged >60 years comprised 31.2% of the 44,672 confirmed cases and 81% of the total number of deaths [13].

To date, no specific treatments or vaccines for COVID-19 have been made available; thus, it is of great importance for older people to engage in preventive behaviors, which has been proved to be effective in preventing respiratory infectious diseases and controlling their transmission [14,15]. It is believed that the transmission route of COVID-19 is human-to-human via respiratory droplets or direct contacts [16] and that the average infected person can spread the disease to up to 3 other people [17]; thus, effective preventive measures, including handwashing, mask-wearing, household ventilation and disinfection, and reduction of interpersonal contacts by avoiding visiting crowded spaces [18], have been recommended to older people by local Chinese authorities. However, these guidelines would severely hamper many daily activities if implemented at a high level of fidelity; therefore, voluntary compliance is likely to be uneven at best.

In fact, in the early stage of the COVID-19 epidemic, older people underestimated the severity of the epidemic, as it affected a relatively small population. They were reluctant to listen to the authorities, believing that there was no need to practice the suggested preventive behaviors for COVID-19. A recent study found that compared with younger adults, older men were less worried about COVID-19 and adopted fewer preventive behaviors [19]. Hence, special efforts should be made to enhance older people’s protection against this new infectious disease.

A good understanding of underlying factors that encourage older people to adopt particular preventive behaviors is significant to provide necessary strategies to implement. A higher level of knowledge has been proved to be associated with positive behavior changes [20,21]. In addition, studies on individuals’ behavior changes toward infectious diseases have suggested that perceptions or beliefs about an outbreak are important in determining the adoption of particular preventive behaviors [22-24]. In particular, the health belief model (HBM) [25] has provided a theoretical framework in previous research for understanding and explaining various health-promoting behaviors [26-28], including preventive behaviors during global pandemics [29-31]. The HBM model reveals that significant perception factors include perceived severity, perceived susceptibility, and perceived benefits. Additionally, previous literature has suggested that self-efficacy in preventing disease [32] and belief in the ability of the government to contain the spread of the epidemic [33] are significantly associated with the practice of preventive measures.

Studies have investigated behavioral responses toward COVID-19 among the general population [34,35] and in specific populations such as health care workers [36] and college students [37,38]; however, literature reports focusing on behavior changes in older people remain scant in spite of the detection of increased vulnerability among this population. Therefore, this paper aims to assess the knowledge, perceived beliefs, and preventive behaviors of older Chinese residents toward COVID-19 and to explore the knowledge, perceived beliefs, and other relevant factors that influence behavior changes. Our findings may help enhance the effectiveness of intervention programs targeting the older population.

## Methods

### Study Design

This cross-sectional study was conducted using an anonymous web-based questionnaire between February 19 and March 19, 2020. A convenience sampling method was used to recruit participants. Chinese residents aged ≥60 years without intellectual or cognitive impairment who agreed to participate in our survey were considered eligible. Those who were illiterate or unable to use electronic devices could ask others to help them fill out the questionnaire; however, it was emphasized that the answers must always reflect the older person’s own opinions. The link to the web-based questionnaire was sent through WeChat (which is similar to WhatsApp) and other social media platforms to the contacts of potential respondents. A brief introduction was presented at the beginning of the survey to inform the respondents of the purpose and content of this study and to instruct them on how to complete the questionnaire. Respondents who completed the questionnaire were entered in a draw for a monetary incentive of approximately ¥1-5 (US $0.15-0.76).

### Sampling

According to Kendall, multiple regression analysis demands a sample size of at least 5 to 10 times the number of independent variables. Thus, we required a maximum sample size of 670 cases, as 67 independent variables were included in this study. Returned surveys with unreasonably short answer times, incomplete information, or logic problems were deemed invalid. The effective sample size estimated for the study was 957 cases, with an invalid response rate of 30%. Finally, a total of 1501 questionnaires were returned in the study duration; after excluding 212 invalid questionnaires and 26 participants who reported having never heard about COVID-19, a total of 1263 surveys (84.1%) from all of the 31 provinces, municipalities, and autonomous regions in mainland China remained for statistical analysis.

### Measures

#### Dependent Variable (Practice of Preventive Behaviors)

The questionnaire on the practice of COVID-19 preventive behaviors was developed based on the guidelines from the Chinese Center for Disease Control and Prevention (CDC). This section contained 5 items, including washing hands frequently, wearing a face mask in public areas, disinfecting one’s household, ventilating one’s household, and avoiding crowds (P1-P5). Answers of “more,” “as usual,” and “less” were rated as 3, 2, and 1, respectively. Therefore, the total behavior score could range from 5 to 15, with a higher score indicating better adherence to preventive behaviors.

#### Independent Variables

##### Background Variables

Sociodemographic characteristics were surveyed in this section of the survey, including gender, age, marital status, education level, registered residential area, and monthly household income per capita. Additionally, the respondents’ self-perceived health status, current addresses at the district level, and local community-level control measures (free entry/exit as usual, entry/exit control exercised, and lockdown) in their residential areas were surveyed. The provinces of the respondents’ current addresses were divided into eastern, central, and western regions according to the National Bureau of Statistics of China and were also categorized into three levels according to their numbers of confirmed COVID-19 cases; provinces with <100 cumulative confirmed cases, 100-1000 cases, and >1000 cases were rated as low, medium, and high-risk areas, respectively.

##### COVID-19–Related Knowledge

The participants’ levels of knowledge about COVID-19 were assessed by 7 questions (K1-K7): 1 regarding the province in which the first COVID-19 cases were reported, 1 regarding the incubation period, 1 regarding the source of infection, 2 regarding transmission modes, 1 regarding susceptible populations, and 1 regarding the availability of a vaccine for COVID-19. The answers were judged according to the guidelines for clinical management of COVID-19 by the National Health Commission of the People’s Republic of China [39]. A correct answer was assigned 1 point, and an incorrect or unknown answer was assigned 0 points. The total knowledge score thus ranged from 0 to 7, with a higher score representing better knowledge of COVID-19.

##### Perceived Beliefs Regarding COVID-19

Based on the HBM and previous studies, the perceived beliefs measured in our study included constructs of HBM (perceived severity, perceived susceptibility, and perceived benefits), self-efficacy in preventing the disease, and belief that the government can contain the spread of the epidemic (B1-B5). Perceived severity of COVID-19 was assessed by 1 item asking the respondents whether they would suffer from more severe symptoms if they contracted the disease; an answer of “yes” was coded as 1, while other answers were coded as 0. One item assessed perceived susceptibility by measuring the degree to which the respondents perceived themselves to be vulnerable to COVID-19; the responses were coded into two groups (1: high/very high; 0: low/very low/I don’t know). Perceived benefits were assessed by three items measuring the respondent’s belief in the effectiveness of COVID-19–related preventive measures of mask wearing in public areas, handwashing, and avoidance of visiting crowded places, respectively; answers of “yes” were considered appropriate (1: yes; 0: others), and the perceived benefits indicator was established by counting the number of appropriate answers (values of 0-3). The respondents’ self-efficacy in preventing the disease and confidence in the government to contain the spread of the epidemic were assessed by asking “Are you confident that you can protect yourself from contracting COVID-19?” and “Do you believe that the government can win the battle against COVID-19?” Answers of “yes” were considered positive and were given 1 point, while 0 points were given for choosing other answers.

### Statistical Analysis

Descriptive statistics were used to summarize the background factors and COVID-19 knowledge, perceived beliefs, and behaviors; the results are presented as frequencies (n) and percentages (%) or as means and SDs. Associations between background variables and behavior scores were examined by one-way analysis of variance (ANOVA) or independent sample *t* tests as appropriate. A multiple linear regression model using statistically significant background variables, knowledge scores, and factors of perceived beliefs as independent variables and scores of preventive behavior as outcome variables was applied to identify factors associated with preventive behaviors. The demographic variables of the respondents were entered in the regression model first to control for their effects. Then, the knowledge score was entered in the next block of the regression, and factors of perceived beliefs were simultaneously entered in the last block. Unstandardized regression coefficients (*β*) and 95% CIs are reported. All statistical analyses were performed in SPSS version 21.0 (IBM Corporation), and a *P* value <.05 was considered statistically significant.

### Ethics Statement

The study protocol was approved by the Ethics Committee of School of Public Health, Zhejiang University (approval number: ZGL202002-2) before the formal survey. The questionnaire was designed to be anonymous and voluntary, and respondents were informed that submission of the questionnaire implied informed consent. The data were kept confidential, and the results did not identify the respondents personally.

## Results

### Background Characteristics

Of the 1263 participants, 730 (57.8%) completed the questionnaire with the help of others, and 687 (54.4%) were registered permanent residents of rural areas. The mean age was 69.48 years (SD 6.72), over half of the respondents were female (697/1263, 55.2%), and approximately three-fourths were married or cohabitating (941/1263, 74.5%). Of the 1263 participants, 586 (46.4%) had an education level of primary school or below, and most respondents (844, 66.8%) had an average household income of between ¥600 and ¥6000 per month (US $91.61-$916.07). Moreover, 564/1263 participants (44.7%) self-reported their physical health status to be fair; 613 (48.5%) of the 1263 respondents lived in the eastern region, and nearly half of the participants (591/1263, 46.8%) lived in areas with medium risk of COVID-19. The majority of respondents (1050/1263, 83.1%) reported that entry and exit control was exercised in their community or village.

### Levels of COVID-19–Related Knowledge, Perceived Beliefs, and Behaviors

#### Knowledge

The mean knowledge score was 6.06 (SD 0.03, range 1-7), suggesting an overall 87% (6.06/7*100) correct rate on this knowledge test (Table 1). Of the 1263 respondents, 85.4% (n=1078) agreed that the current main source of infection was patients with COVID-19. In terms of modes of transmission, 98.1% of respondents (1239/1263) thought that COVID-19 can be transmitted by droplets emitted by patients; however, less than 80% (960/1263, 76.0%) were aware that it can also be transmitted through virus-contaminated objects. Approximately 90% of respondents (1165/1263, 92.2%) correctly stated that the population is generally susceptible to the virus regardless of age. However, only 64.1% of the respondents (809/1263) knew that effective vaccines for COVID-19 were not yet available*.*

**Table 1:** Participants’ knowledge and perceived beliefs regarding COVID-19 (N=1263).

Item			Response
**Knowledge items**			
	**K1: The earliest outbreak of COVID-19 in China occurred in Hubei Province, n (%)**		
		Correct	1258 (99.6)
		Incorrect/unknown	5 (0.4)
	**K2: The incubation period of COVID-19 is 1-14 days, n (%)**		
		Correct	1139 (90.2)
		Incorrect/unknown	124 (9.8)
	**K3: Currently, the main source of infection is patients with COVID-19, n (%)**		
		Correct	1078 (85.4)
		Incorrect/unknown	185 (14.6)
	**K4: COVID-19 can be transmitted by droplets from a patient, n (%)**		
		Correct	1239 (98.1)
		Incorrect/unknown	24 (1.9)
	**K5: COVID-19 can be transmitted through touching virus-contaminated surfaces, n (%)**		
		Correct	960 (76.0)
		Incorrect/unknown	303 (24.0)
	**K6: All age groups can become infected with the new coronavirus, n (%)**		
		Correct	1165 (92.2)
		Incorrect/unknown	98 (7.8)
	**K7: There is currently no vaccine available that protects against COVID-19, n (%)**		
		Correct	809 (64.1)
		Incorrect/unknown	454 (35.9)
	Range of knowledge scores		1-7
	Mean knowledge score (SD)		6.06 (0.03)
**Perceived belief items, n (%)**			
	**B1: Perceived severity of COVID-19**		
		Yes	1141 (90.3)
		No/I don’t know	122 (9.7)
	**B2: Perceived susceptibility of COVID-19**		
		High/very high	190(15.0)
		Low/very low/I don’t know	1073(85.0)
	**B3: Perceived benefits indicator^a^**		
		0	6 (0.5)
		1	26 (2.1)
		2	68 (5.4)
		3	1163 (92.1)
	**B4: Self-efficacy in preventing COVID-19**		
		Yes	1048 (83.0)
		No/I don’t know	215 (17.0)
	**B5: Confidence in the government to control the spread of COVID-19**		
		Yes	1160 (91.8)
		No/I don’t know	103 (8.2)

^a^The perceived benefits indicator was established by counting the number of items with “yes” responses for the three basic preventive measures (ie, handwashing, face mask wearing, and staying at home).

#### Perceived Beliefs

The majority of the older respondents (1141/1263, 90.3%) believed that older people may suffer from more severe symptoms if they are infected with COVID-19 (Table 1). Most respondents (1073/1263, 85.0%) did not perceive that their likelihood of contracting COVID-19 was high or very high. A substantial proportion of participants (1163/1263, 92.1%) perceived the benefits of all three types of preventive measures (ie, handwashing, wearing a face mask, and staying at home). Additionally, 83.0% of the respondents (1048/1263) were confident in their own abilities to prevent the disease, and 91.8% (1160/1263) believed that the government can “win the battle” against COVID-19.

#### Behaviors

The mean score of preventive behavior toward COVID-19 was 13.73 (SD 1.62, range 7-15). The participants’ preventive behavior changes in response to the COVID-19 pandemic are shown in Table 2. It is reassuring that more than four-fifths of our 1263 participants had increased their frequency of handwashing (n=1025, 81.2%) and wearing a face mask in public venues (n=1035, 81.9%) to ensure their safety; in addition, 86.6% (n=1094) of the older people had reduced their visits to crowded places. However, fewer participants had increased their household ventilation (956/1263, 75.5%) and disinfection (816/1263, 64.6%).

**Table 2:** Participants’ preventive behavior changes in response to the COVID-19 pandemic in the past week (N=1263), n (%). Questions were answered using the following scale: 1=less, 2=as usual, and 3=more. For the total behavior score, we calculated how many specific behavior changes each participant endorsed. The range of the behavior scores was 7-15, and the mean score was 13.73 (SD 1.62).

Behavior	More	Less	As usual
P1: Face mask wearing in public venues	1035 (81.9)	60 (4.8)	168 (13.3)
P2: Washing of hands	1025 (81.2)	12 (1.0)	226 (17.9)
P3: Household ventilation	956 (75.5)	66 (5.2)	241 (19.1)
P4: Home disinfection	816 (64.6)	38 (3.0)	409 (32.4)
P5: Avoidance of crowds	1094 (86.6)	37 (2.9)	132 (10.5)

### Demographic Factors of Preventive Behaviors: Univariate Analysis

As shown in Table 3, region, marital status, education level, residence registration, and monthly household income per capita were significantly associated with preventive behaviors (all *P*<.05). Participants who lived in western regions had significantly lower level of behavior than participants from eastern regions (*P*=.01). Respondents who were married or cohabitating had significantly higher behavior scores (*P*<.001). Rural dwellers and respondents who attended primary school or below had lower behavior scores (both *P*<.001). The behavior scores of participants with an average household income of <¥600 per month (US $91.61) were significantly lower than those of participants with monthly incomes higher than ¥6000 (US $916.07; *P*=.02). Interestingly, a significant association between local community-level control measures and behavior score was observed (*P*<.001); respondents engaged in more preventive behaviors when epidemic control measures were exercised in their areas of residence.

**Table 3:** Univariate analysis of the demographic variables associated with preventive behaviors (N=1263).

Variable		Participants, n (%)	Behavior score^a^		
			Mean (SD)	*t*/*F* (df)	*P* value
**Gender**				*t_1262_*=0.208	.65
	Male	566 (44.8)	13.71 (1.63)		
	Female	697 (55.2)	13.75 (1.61)		
**Age (years)**				*F_(2,1260)_*=2.925	.054
	60-69	683 (54.1)	13.81 (1.61)		
	70-79	474 (37.5)	13.69 (1.62)		
	≥80	106 (8.4)	13.42 (1.67)		
**Marital status^b^**				*t_1262_*=18.625	*<.001^c^*
	Married/cohabiting	941 (74.5)	13.85 (1.56)		
	Single/divorced/ separated/widowed	322 (25.5)	13.40 (1.74)		
**Education level^b^**				*F_(3,1259)_*=8.904	*<.001*
	Primary education or below	586 (46.4)	13.50 (1.67)^d^		
	Middle school	330 (26.1)	13.94 (1.57)^e^		
	High school	197 (15.6)	13.79 (1.65)^e^		
	College or above	150 (11.9)	14.11 (1.33)^e^		
**Registered residential area^b^**				*t_1262_*=20.090	*<.001*
	Urban	576 (45.6)	13.95 (1.52)		
	Rural	687 (54.4)	13.55 (1.68)		
**Monthly household income per capita (¥)^b,f^**					
	＜600	219 (17.3)	13.51 (1.78)^d^	*F_(2,1260)_*=4.031	*.02*
	600-6000	844 (66.8)	13.74 (1.63)^d,e^		
	＞6000	200 (15.8)	13.96 (1.36)^e^		
**Self-reported health status**				*F_(4,1258)_*=0.375	.83
	Excellent	57 (4.5)	13.72 (1.68)		
	Very good	220 (17.4)	13.85 (1.58)		
	Good	378 (29.9)	13.68 (1.73)		
	Fair	564 (44.7)	13.72 (1.57)		
	Poor	44 (3.5)	13.75 (1.53)		
**Region^b^**				*F_(2,1260)_*=4.393	*.01*
	Eastern	613 (48.5)	13.86 (1.49)^d^		
	Central	183 (14.5)	13.72 (1.64)^d,e^		
	Western	467 (37.0)	13.57 (1.77)^e^		
**Provincial COVID-19 epidemic level^g^**				*F_(2,1260)_*=1.166	.31
	Low risk: <100 cases	146 (11.6)	13.73 (1.68)		
	Medium-risk: 100-999 cases	591 (46.8)	13.66 (1.69)		
	High risk: ≥1000 cases	526 (41.6)	13.81 (1.52)		
**Local community-level control measures^b^**				*F_(2,1260)_*=7.634	*<.001*
	Entry/exit control exercised	1050 (83.1)	13.70 (1.63)^d^		
	Lockdown	181 (14.3)	14.04 (1.45)^d^		
	Free entry/exit as usual	32 (2.5)	12.91 (1.91)^e^		

^a^Values based on independent sample *t* tests and one-way analysis of variance (ANOVA) for continuous variables to examine differences between preventive behaviors and demographic variables. For categories of variables with significant ANOVA results (significant at *P*<.05 between the groups), multiple comparisons between each of 2 categories were performed by post hoc analysis (least significant difference).

^b^Significant variables were included in the subsequent multivariable analyses.

^c^Italics indicate statistical significance.

^d,e^Within each column, if two means share same superscript (d or e), they are not statistically different (*P*>.05) from one another.

^f^1 ¥=US $0.14 on February 20, 2020.

^g^Provincial COVID-19 epidemic level: The number of cumulative confirmed COVID-19 cases on the survey launch day (ie, March 20, 2020) in the province where the respondent was located during the COVID-19 pandemic.

### Factors Associated With Behaviors: Multivariable Analyses

The knowledge scores and perceived belief-related variables reported in Table 1, together with significant background variables, were included in the hierarchical linear regression models. In block 1, the demographic factors of region, marital status, education level, registered residence, monthly household income per capita, and local community-level control measures were entered first, accounting for 4.7% of the variance (*R^2^*=0.047, *F*_6,1256_=5.575, *P*<.001). The score for knowledge about COVID-19 was entered into block 2 and contributed an additional 2.9% of the variance (*R^2^*=0.076, *F*_7,1255_=8.603, *P*<.001). Finally, factors of perceived beliefs regarding COVID-19, including perceived severity, perceived susceptibility, perceived benefits, self-efficacy in preventing the disease, and confidence in the government, were entered in the third block; these factors were significant in predicting behaviors even after considering the effects of background and knowledge factors, and they contributed an additional 3.2% of the variance (*R^2^*=0.108, *F_12,1250_*= 8.904, *P*<.001). The linear regression analysis of the preventive behaviors is presented in Table 4 and Table 5.

Education level and registered residence were generally nonsignificant, while some significant marital status and control measure differences were noted. Overall, respondents who were married or cohabitating (vs single, divorced, separated, or widowed, *β*=0.355, 95% CI 0.150-0.560) and who could not leave the house (vs free entry and exit, *β*=0.898, 95% CI 0.310-1.486) were more likely to have better preventive behavior scores (*P*<.01). After adjusting for background characteristics, we discovered that older people with higher COVID-19–related knowledge scores had significantly higher behavior scores (*β*=0.198, *P*<.001). Meanwhile, the results showed that 3 of the 5 components of perceived beliefs, namely, perceived susceptibility (*β*=0.263, 95% CI 0.022-0.504), perceived benefits (*β* =0.643, 95% CI 0.305-0.982), and self-efficacy in preventing the disease (*β* =0.468, 95% CI 0.223-0.713), were positively associated with preventive behaviors (all *P*<.05); however, perceived severity and confidence in the government were not significant predictors of preventive behaviors. Among the influencing factors, local community-level control measures showed the greatest impact on behaviors, followed by perceived benefits.

**Table 4:** Multivariable analysis of factors associated with preventive behaviors (N=1263).

Characteristic			Model 1		Model 2		Model 3	
			*β* (95% CI)	*P* value	*β* (95% CI)	*P* value	*β* (95% CI)	*P* value
**Block 1: Background characteristics**								
	**Region**							
		Western	Reference	N/A^a^	Reference	N/A	Reference	N/A
		Eastern	0.156 (0.049 to 0.361)	.14	0.082 (–0.121 to 0.285)	.43	0.085 (–0.116 to 0.286)	.41
		Central	0.087 (–0.186 to 0.360)	.53	0.013 (–0.256 to 0.283)	.92	–0.051 (–0.318 to –0.217)	.71
	**Marital status**							
		Single/divorced/separated/widowed	Reference	N/A	Reference	N/A	Reference	N/A
		Married/cohabitating	0.330 (0.120 to 0.541)	*.002^b^*	0.331 (0.124 to 0.538)	*.002*	0.355 (0.150 to 0.560)	*<.001*
	**Education level**							
		Primary education or below	Reference	N/A	Reference	N/A	Reference	N/A
		Middle school	0.244 (0.014 to 0.475)	*.04*	0.177 (–0.050 to 0.405)	.13	0.137 (–0.088 to 0.362)	.23
		High school	–0.002 (–0.293 to 0.290)	.99	–0.106 (–0.396 to 0.183)	.47	–0.117 (–0.402 to 0.168)	.42
		College or above	0.258 (–0.078 to 0.594)	.13	0.123 (–0.210 to 0.457)	.47	0.096 (–0.233 to 0.425)	.57
	**Registered residential area**							
		Rural	Reference	N/A	Reference	N/A	Reference	N/A
		Urban	0.243 (0.031 to 0.455)	*.02*	0.184 (0.025 to 0.393)	.09	0.122 (0.085 to 0.329)	.25
	**Monthly household income per capita (¥)^c^**							
		＜600	Reference	N/A	Reference	N/A	Reference	N/A
		600-6000	0.047 (–0.203 to 0.297)	.71	0.031 (–0.215 to 0.422)	.81	0.018 (–0.226 to 0.261)	.89
		＞6000	0.113 (–0.229 to 0.454)	.52	0.086 (–0.251 to 0.422)	.62	0.067 (–0.266 to 0.399)	.70
	**Local community-level control measures**							
		Free entry/exit as usual	Reference	N/A	Reference	N/A	Reference	N/A
		Entry/exit control exercised	0.671 (0.108 to 1.234)	*.02*	0.635 (0.081 to 1.190)	*.03*	0.501 (–0.049, 1.051)	.07
		Lockdown	1.062 (0.460 to 1.663)	*<.001*	1.034 (0.441 to 1.627)	*<.001*	0.898 (0.310 to 1.486)	*.003*
**Block 2: Knowledge**								
	Knowledge related to COVID-19		—^d^	—	0.270 (0.186 to 0.353)	*<.001*	0.198 (0.111 to 0.286)	*<.001*
**Block 3: Perceived beliefs**								
	Perceived severity		—	—	—	—	–0.045 (–0.350 to 0.259)	.77
	Perceived susceptibility		—	—	—	—	0.263 (0.022 to 0.504)	*.03*
	Perceived benefits indicator^e^		—	—	—	—	0.643 (0.305 to 0.982)	*<.001*
	Self-efficacy in preventing COVID-19		—	—	—	—	0.468 (0.223 to 0.713)	*<.001*
	Confidence in the government to fight COVID-19		—	—	—	—	0.321 (–0.014 to 0.656)	.06

^a^N/A: not applicable.

^b^Italics indicate statistical significance between groups.

^c^1 ¥=US $0.14 on February 20, 2020.

^d^—: Data not included in this model.

^e^The perceived benefits indicator was established by counting the number of items with “yes” responses for the three basic preventive measures (ie, handwashing, face mask wearing, and staying at home).

**Table 5:** Statistical measures of the 3 models of the multivariable analysis (*P*<.001 for all models).

Measure	Model 1	Model 2	Model 3
*F*	5.575	8.603	8.904
*R* ^2^	.047	.076	.108
*Δ* *R* ^2^	.047	.029	.032
Adjusted *R*^2^	.038	.067	.096

## Discussion

### Principal Findings

Older people, who are particularly vulnerable to acute diseases and their complications, are currently greatly threatened by the outbreak of COVID-19. Special effort should therefore be made to encourage older people to practice the behaviors suggested by the government to prevent the disease. An understanding of older people’s knowledge, perceived beliefs, and behaviors toward COVID-19, which affect the adoption of related health behaviors, can be a first step to prevent the spread of COVID-19 in this population.

In this study, it was found that Chinese older adults had good knowledge of COVID-19; the respondents had an overall correct rate of 87% on the knowledge questionnaire, which was slightly lower than a rate reported for the Chinese general population (90%) [40] but much higher than that reported for US residents (80%) [41]. The high correct rate was surprising, as previous studies suggested that knowledge scores are negatively related with age [42-44]. However, this finding may be due to the large amounts of publicity related to COVID-19 through various channels that are appropriate to the needs and characteristics of older people, such as vivid prints, marked banners, and broadcasts in dialect. The 2 items with the lowest correct rates are worth mentioning. Knowledge about modes of transmission, which has been proved to be a salient factor influencing the level of adoption of preventive measures [45], should be further improved among older people. Meanwhile, older people should also be informed that an effective vaccine for COVID-19 is not yet available and that taking precautions against the disease is still highly important.

The perceived beliefs of older Chinese residents toward COVID-19 were found to be optimistic overall. Although the majority of respondents (1141/1263, 90.3%) perceived that older adults would suffer from more severe symptoms if they were infected, relatively few respondents (190/1263, 15.0%) thought they were at high risk of acquiring the disease. Moreover, the majority expressed confidence in themselves (1048/1263, 83.0%) and the government (1160/1263, 91.8%) to stop the spread of COVID-19. This optimistic belief could be explained by the unprecedented epidemic control measures taken by the Chinese government after the outbreak of COVID-19 in the country and the concerted efforts of people all over China to prevent the spread of the disease. From January 20, 2020, when person-to-person transmission was confirmed and the Chinese public was notified of this finding, a series of nationwide public health emergency measures, including isolation and quarantine, close management of working and living spaces, and the Examine and Approve Policy on the resumption of work, were implemented by the Chinese government, health institutions, communities, companies, etc [46].

In addition to efforts from all sectors of society, high adherence to the lifestyle modifications suggested by the government and public health organizations greatly delayed the spread of the disease [47]. In our findings, 64.6%-86.6% of respondents reported practicing five major preventive behaviors more frequently in the preceding week. The reason for this finding may be that this study was conducted at a time when these key preventive measures were highly emphasized in China. Similarly high tendencies to adopt these precautionary behaviors during the pandemic have been found in other studies [35,48]. In particular, more than 80% of respondents reported increased handwashing and face mask wearing and decreased time spent in crowds, indicating that basic protective behaviors against COVID-19 had taken root among older people. The majority of participants avoided going out, which can potentially be attributed to the widely disseminated governmental propaganda and social media messages, which continuously instilled and reinforced people’s incentives to stay home (eg, personal safety, good citizenship, and contribution to the control of the national epidemic). Moreover, individuals may have felt pressure from the community to adhere to behaviors such as imposed lockdowns or quarantine of entire regions with suspected cases. Handwashing and wearing of face masks have long been regarded as significant preventive habits in the daily life of the public since they were proved to be efficacious in preventing influenza and SARS [49,50]. Lau et al [51] observed that hand-washing and face mask wearing were commonly practiced during the SARS outbreak, and these behaviors were sustained by a large proportion of the public even after the SARS epidemic subsided in Hong Kong. In contrast, the effectiveness of household ventilation and disinfection may have been neglected, with fewer participants reporting that they had practiced these two behaviors more frequently in the past week. Habit has a great impact on routine behavior—including hygiene behavior—and despite their best intentions, people may find it difficult to implement new measures during a pandemic if they were not previously in the habit of performing them [52,53]. Further education on practicing household ventilation and disinfection is needed, as these two measures are indispensable in preventing COVID-19.

In line with previous research [54-56], our study also found that individuals’ demographic characteristics had a significant influence on their preventive behaviors. Linear regression analysis of the behavior scores showed that participants who were married or cohabitating were more likely to report that they had complied with advocated protective behaviors more frequently; this finding was consistent with similar studies regarding SARS [57,58]. On the one hand, older adults who are married or cohabiting can receive support from their spouse or partner. Family support has been shown to influence older people’s health beliefs and self-care behaviors [59]. The more family support older people receive, the more attention they will pay to their own health, accompanied with increased willingness to acquire health knowledge and more positive preventive beliefs. On the other hand, according to the HBM, advice from family members can be regarded as an external cue to action, which is also a very important factor in increasing various preventive behaviors [60]. Consistent with research by Kong et al [48], age was not a significant factor of preventive behaviors in our study (*P*=.054); meanwhile, other studies have found that compared with younger participants, individuals aged 60 years or older implemented fewer preventive behavior changes [19,61]. Further larger-scale studies are warranted to assess the association between age and behaviors to prevent COVID-19.

Additionally, community-level control measures were found to be significantly associated with the practice of preventive measures. As COVID-19 rapidly spread from a single city to the entire country, the governmental “minimum contact strategy” was implemented nationwide; also, close management and screening of communities and villages to curb COVID-19 were subsequently implemented nationally in China [46]. Although the contribution of community control measures cannot be quantified, our study supports that community-level control measures against COVID-19 are related to significant increases in older adults’ practice of recommended preventive behaviors. We further confirmed that respondents who could not leave the house showed higher compliance with suggested preventive measures than those who could move freely as usual. According to the socioecological model, factors of structural, interpersonal, and personal levels determine health-related behaviors [62]. At the personal level, it has been found that older Chinese people feel they have an ethical duty (“filial piety”) to protect others, which facilitates their adherence to quarantine. In Wenzel’s study [63], “filial piety” suggested the ‘‘right and humane’’ way to act toward one’s family and others in the community and guided Chinese older adults in their use of strategies against SARS [63]. As social beings, people’s behaviors are subject to the influence of social relationships [64]. Chinese older people not only worry for themselves but also have concern for their significant others regarding COVID-19, which motivates their adoption of precautionary measures. Meanwhile, environmental manipulation and policies are crucial structural-level factors to encourage individuals to practice desired preventive behaviors. According to the WHO, social distancing/self-isolation and lockdown are two important nationwide social measures during a public health crisis [6]. The Chinese government adopted these strategies to mitigate the risk of COVID-19. Therefore, personal perceived ethical duty combined with community actions have facilitated China’s response to COVID-19. Particularly, community and home quarantine provided Chinese older adults with ample time to communicate with and influence their significant others. The abovementioned findings highlight the importance of control measures at the community level, which should be considered when planning pandemic control strategies in the future.

In addition to background factors, this study found that knowledge and perceived beliefs toward COVID-19 were significant predictors of preventive behaviors. In accordance with previous findings, this study confirmed that people with adequate knowledge about COVID-19 were more likely to take preventive measures in response to the pandemic than people who lacked such knowledge [56,65]; this suggests that health education aimed at improving people’s COVID-19 knowledge plays an important role in promoting preventive behaviors toward COVID-19. With regard to perceived belief–related factors, our study confirmed the positive correlation between self-efficacy in preventing COVID-19 and the adoption of preventive measures found in previous research [32,54]; however, bidirectional effects may exist between these two variables, as it has been suggested that perceived self-efficacy can encourage an individual to adopt certain behaviors to achieve desired outcomes, while success in performing certain behaviors can further augment perceived self-efficacy [66]. Contrary to previous findings [22,30,33], our study found that belief in the efficacy of local health authorities did not significantly predict the target COVID-19 preventive behaviors. The belief in government may not successfully translate into positive behavioral changes at the individual level; additionally, the positive relationship between trust in the government and the practice of preventive behaviors can be mediated by levels of perceived self-efficacy [20]. A relatively strong linear correlation between confidence in the government and self-efficacy (*r*=0.335, *P*<.001) was also found in this study.

Some of the items we used to assess the participants’ perceived beliefs were derived from the HBM, which includes perceived severity, perceived susceptibility, and perceived benefits. Although most of our respondents perceived that they might progress to serious symptoms if they were infected with COVID-19, perceived severity was not found to be significantly related to COVID-19–related preventive behaviors in this study. Perceived severity has been suggested to have relatively low relevance for preventive health behaviors; however, it may play an important role when individuals have already been diagnosed with certain diseases [25]. Therefore, the fact that the participants had not yet been diagnosed with COVID-19 as well as their low perceived susceptibility could account for the insignificant association between perceived severity and preventive behaviors. Among our participants, perceived susceptibility was found to be a significant correlate of COVID-19 preventive health behaviors. Perceived susceptibility has been consistently found to be a salient determinant of participation in preventive measures during an epidemic among both the general population and older people [23,33,67,68]. Other studies on health screening [69,70] and exercise behavior [71] also found that perceived susceptibility plays an important role in participation in protective behaviors. Despite the significant positive association between perceived susceptibility and engagement in protective behaviors, only 15.0% (190/1263) of the older people in this study perceived that they were highly vulnerable to COVID-19. This finding indicates that older people should be further warned about their higher vulnerability to the disease; moreover, to ensure the effectiveness of warnings, messages issued by public health communicators should be comprehensible, concise, and convincing [22]. Meanwhile, perceived benefits were found to be more prominent in affecting individuals’ adoption of preventive behaviors in the aforementioned HBM constructs [26]. When people deem certain preventive health behaviors to be effective in preventing a disease, they are motivated to engage in these behaviors [22,58,72,73]. Indeed, communications that increase individuals’ perceptions of the benefits of particular health-related behaviors have been proved to be successful in reducing health threats [74-76]. Health authorities should be reminded to exert effort to provide sufficient health communication messages to enhance older people’s perceived susceptibility and benefits in the context of the COVID-19 pandemic, which can greatly motivate these individuals to engage in preventive measures.

### Limitations

Our study has some limitations. First, no standardized tool for assessing knowledge, perceived beliefs, or practice of preventive behaviors on COVID-19 has been previously validated. We designed the questionnaire based on the latest official report from the WHO, the Chinese CDC, and the scientific literature; however, the depth of the survey may be limited, as we were obligated to conduct our investigation quickly and recruit as large a sample as possible. Secondly, selection bias may exist. The web-based survey only included people who have access to the internet, and older people who were infected or had close contacts with confirmed or suspected COVID-19 patients may not have wanted to participate in this survey; thus, the generalizability of our findings may be limited. However, web-based surveys may be the most appropriate method of data collection during an epidemic, as this method can prevent transmission; moreover, fielding the survey offline was not feasible, as strict epidemic control measures were being exercised in most parts of the country. Thirdly, this study was cross-sectional, and no casual effect statements concerning the relationship between targeting variables and the performance of preventive behaviors could be made. Fourthly, recall and social desirability bias may exist, as only retrospective self-reports of the participants were collected.

### Strengths

Despite the above limitations, this is one of few studies to assess the knowledge, perceived beliefs, and preventive behaviors toward COVID-19 among the older adult population in China. Meanwhile, a large number of respondents from all provinces in mainland China were recruited, enabling us to obtain a wide range of participants with various demographic backgrounds. Additionally, the investigation was conducted in the stable epidemic period (February 19 to March 19, 2020), when community-wide COVID-19 prevention activities were launched by local health authorities; thus, we were able to assess how community-level control measures influenced personal preventive behaviors. Therefore, our results are of practical significance for the design and implementation of health programs for the older population in the prevention of COVID-19 and other emerging epidemics.

### Conclusions

Generally, the Chinese older adults in our survey demonstrated good knowledge, optimistic perceived beliefs, and appropriate behaviors toward COVID-19 during the pandemic, which are important factors to limit the spread of the disease. Higher behavior scores were found among older adults who were married and cohabitating and who were restricted by community-level control measures, suggesting that health education programs should pay more attention to people who are single and can move around freely. In addition, our findings suggest that good knowledge and appropriate perceived beliefs are associated with high levels of engagement in behaviors to prevent COVID-19. Therefore, to promote preventive behaviors, continued health intervention programs are advised among older people to improve their knowledge in certain aspects, including transmission modes and vaccines against COVID-19; additionally, older people’s perception of their own vulnerability to COVID-19 and the effectiveness of COVID-19–related preventive behaviors should be further emphasized.

## Abbreviations

ANOVA: analysis of variance
CDC: Center for Disease Control and Prevention
HBM: health belief model
MERS: Middle East respiratory syndrome
SARS: severe acute respiratory syndrome
WHO: World Health Organization

## References

[1] (2020). Coronavirus disease (COVID-19) Situation Report – 202. World Health Organization.

[2] Munster VJ, Koopmans M, van Doremalen N, van Riel D, de Wit E (2020). A Novel Coronavirus Emerging in China - Key Questions for Impact Assessment. N Engl J Med.

[3] Zhou T, Liu Q, Yang Z, Liao J, Yang K, Bai W, Lu X, Zhang W (2020). Preliminary prediction of the basic reproduction number of the Wuhan novel coronavirus 2019-nCoV. J Evid Based Med.

[4] Meo SA, Alhowikan AM, Al-Khlaiwi T, Meo IM, Halepoto DM, Iqbal M, Usmani AM, Hajjar W, Ahmed N (2020). Novel coronavirus 2019-nCoV: prevalence, biological and clinical characteristics comparison with SARS-CoV and MERS-CoV. Eur Rev Med Pharmacol Sci.

[5] 2019-nCo VIAEOIC (2020). 2019-nCoV outbreak is an emergency of international concern. World Health Organization.

[6] (2020). WHO Director-General's opening remarks at the media briefing on COVID-19. World Health Organization.

[7] Rajagopalan S (2001). Tuberculosis and aging: a global health problem. Clin Infect Dis.

[8] Nikolich-Zugich J, Knox KS, Rios CT, Natt B, Bhattacharya D, Fain MJ (2020). SARS-CoV-2 and COVID-19 in older adults: what we may expect regarding pathogenesis, immune responses, and outcomes. Geroscience.

[9] Banerjee D (2020). The impact of Covid-19 pandemic on elderly mental health. Int J Geriatr Psychiatry.

[10] Liu K, Chen Y, Lin R, Han K (2020). Clinical features of COVID-19 in elderly patients: A comparison with young and middle-aged patients. J Infect.

[11] Onder G, Rezza G, Brusaferro S (2020). Case-Fatality Rate and Characteristics of Patients Dying in Relation to COVID-19 in Italy. JAMA.

[12] Wang D, Hu B, Hu C, Zhu F, Liu X, Zhang J, Wang B, Xiang H, Cheng Z, Xiong Y, Zhao Y, Li Y, Wang X, Peng Z (2020). Clinical Characteristics of 138 Hospitalized Patients With 2019 Novel Coronavirus-Infected Pneumonia in Wuhan, China. JAMA.

[13] Epidemiology Working Group for NCIP Epidemic Response‚ Chinese Center for Disease Control and Prevention (2020). The epidemiological characteristics of an outbreak of 2019 novel coronavirus diseases (COVID-19) in China. Article in Chinese. Zhonghua Liu Xing Bing Xue Za Zhi.

[14] Visentin LM, Bondy SJ, Schwartz B, Morrison LJ (2009). Use of personal protective equipment during infectious disease outbreak and nonoutbreak conditions: a survey of emergency medical technicians. CJEM.

[15] Seto WH, Tsang D, Yung RWH, Ching TY, Ng TK, Ho M, Ho LM, Peiris JSM, Advisors of Expert SARS group of Hospital Authority (2003). Effectiveness of precautions against droplets and contact in prevention of nosocomial transmission of severe acute respiratory syndrome (SARS). Lancet.

[16] Lai C, Shih T, Ko W, Tang H, Hsueh P (2020). Severe acute respiratory syndrome coronavirus 2 (SARS-CoV-2) and coronavirus disease-2019 (COVID-19): The epidemic and the challenges. Int J Antimicrob Agents.

[17] Alimohamadi Y, Taghdir M, Sepandi M (2020). Estimate of the Basic Reproduction Number for COVID-19: A Systematic Review and Meta-analysis. J Prev Med Public Health.

[18] (2020). COVID-19 Guidance for Older Adults. US Centers for Disease Control and Prevention.

[19] Barber SJ, Kim H (2020). COVID-19 Worries and Behavior Changes in Older and Younger Men and Women. J Gerontol B Psychol Sci Soc Sci.

[20] Liao Q, Cowling B, Lam WT, Ng MW, Fielding R (2010). Situational awareness and health protective responses to pandemic influenza A (H1N1) in Hong Kong: a cross-sectional study. PLoS One.

[21] Kamate SK, Agrawal A, Chaudhary H, Singh K, Mishra P, Asawa K (2009). Public knowledge, attitude and behavioural changes in an Indian population during the Influenza A (H1N1) outbreak. J Infect Dev Ctries.

[22] Rubin GJ, Amlôt Richard, Page L, Wessely S (2009). Public perceptions, anxiety, and behaviour change in relation to the swine flu outbreak: cross sectional telephone survey. BMJ.

[23] Yıldırım M, Geçer E, Akgül Ö (2020). The impacts of vulnerability, perceived risk, and fear on preventive behaviours against COVID-19. Psychol Health Med.

[24] Gesser-Edelsburg A, Cohen R, Hijazi R, Abed Elhadi Shahbari N (2020). Analysis of Public Perception of the Israeli Government's Early Emergency Instructions Regarding COVID-19: Online Survey Study. J Med Internet Res.

[25] Janz NK, Becker MH (1984). The Health Belief Model: a decade later. Health Educ Q.

[26] Carpenter CJ (2010). A meta-analysis of the effectiveness of health belief model variables in predicting behavior. Health Commun.

[27] Jones CJ, Smith H, Llewellyn C (2014). Evaluating the effectiveness of health belief model interventions in improving adherence: a systematic review. Health Psychol Rev.

[28] Gu Y, Li L, Zhou C, Yang T, Dong H (2014). Factors influencing voluntary premarital medical examination in Zhejiang province, China: a culturally-tailored health behavioral model analysis. BMC Public Health.

[29] Wong C, Tang CS (2005). Practice of habitual and volitional health behaviors to prevent severe acute respiratory syndrome among Chinese adolescents in Hong Kong. J Adolesc Health.

[30] Quinn SC, Kumar S, Freimuth VS, Kidwell K, Musa D (2009). Public willingness to take a vaccine or drug under Emergency Use Authorization during the 2009 H1N1 pandemic. Biosecur Bioterror.

[31] Tang CSK, Wong C (2003). An outbreak of the severe acute respiratory syndrome: predictors of health behaviors and effect of community prevention measures in Hong Kong, China. Am J Public Health.

[32] Lau JTF, Yang X, Tsui H, Pang E, Kim JH (2004). SARS preventive and risk behaviours of Hong Kong air travellers. Epidemiol Infect.

[33] Tang CS, Wong C (2005). Psychosocial factors influencing the practice of preventive behaviors against the severe acute respiratory syndrome among older Chinese in Hong Kong. J Aging Health.

[34] Clements JM (2020). Knowledge and Behaviors Toward COVID-19 Among US Residents During the Early Days of the Pandemic: Cross-Sectional Online Questionnaire. JMIR Public Health Surveill.

[35] Niu Z, Wang T, Hu P, Mei J, Tang Z (2020). Chinese Public's Engagement in Preventive and Intervening Health Behaviors During the Early Breakout of COVID-19: Cross-Sectional Study. J Med Internet Res.

[36] Bashirian S, Jenabi E, Khazaei S, Barati M, Karimi-Shahanjarini A, Zareian S, Rezapur-Shahkolai F, Moeini B (2020). Factors associated with preventive behaviours of COVID-19 among hospital staff in Iran in 2020: an application of the Protection Motivation Theory. J Hosp Infect.

[37] Huckins JF, daSilva AW, Wang W, Hedlund E, Rogers C, Nepal SK, Wu J, Obuchi M, Murphy EI, Meyer ML, Wagner DD, Holtzheimer PE, Campbell AT (2020). Mental Health and Behavior of College Students During the Early Phases of the COVID-19 Pandemic: Longitudinal Smartphone and Ecological Momentary Assessment Study. J Med Internet Res.

[38] Zhang M, Li Q, Du X, Zuo D, Ding Y, Tan X, Liu Q (2020). Health Behavior Toward COVID-19: The Role of Demographic Factors, Knowledge, and Attitude Among Chinese College Students During the Quarantine Period. Asia Pac J Public Health.

[39] (2020). Guideline for the diagnosis and treatment of 2019 novel coronavirus (2019-nCoV) infected pneumonia (The Third Trial Version). General Office of the National Health Commission of the People's Republic of China.

[40] Zhong B, Luo W, Li H, Zhang Q, Liu X, Li W, Li Y (2020). Knowledge, attitudes, and practices towards COVID-19 among Chinese residents during the rapid rise period of the COVID-19 outbreak: a quick online cross-sectional survey. Int J Biol Sci.

[41] Clements JM (2020). Knowledge and Behaviors Toward COVID-19 Among US Residents During the Early Days of the Pandemic: Cross-Sectional Online Questionnaire. JMIR Public Health Surveill.

[42] Jehn M, Kim Y, Bradley B, Lant T (2011). Community knowledge, risk perception, and preparedness for the 2009 influenza A/H1N1 pandemic. J Public Health Manag Pract.

[43] Alanzi M, Albalawi M, Kabrah S, Aljehani Y, Okashah A, Aljohani Z, Alribyawi R, Alluqmany A, Alanzi K, Alanazi N, Eltahlawi R, Abdel-Haleem M, El-Dardear A, El Sayed S (2019). Knowledge, Attitudes, and Practices (KAPs) of Healthcare Workers towards MERS-CoV Infection at PHCs in Madinah, KSA during Hajj 1440, 2019. Am J Microbiol Res.

[44] Lin Y, Huang L, Nie S, Liu Z, Yu H, Yan W, Xu Y (2011). Knowledge, attitudes and practices (KAP) related to the pandemic (H1N1) 2009 among Chinese general population: a telephone survey. BMC Infect Dis.

[45] Lau JTF, Griffiths S, Choi KC, Tsui HY (2009). Widespread public misconception in the early phase of the H1N1 influenza epidemic. J Infect.

[46] Huang Y, Wu Q, Wang P, Xu Y, Wang L, Zhao Y, Yao D, Xu Y, Lv Q, Xu S (2020). Measures Undertaken in China to Avoid COVID-19 Infection: Internet-Based, Cross-Sectional Survey Study. J Med Internet Res.

[47] Doogan C, Buntine W, Linger H, Brunt S (2020). Public Perceptions and Attitudes Toward COVID-19 Nonpharmaceutical Interventions Across Six Countries: A Topic Modeling Analysis of Twitter Data. J Med Internet Res.

[48] Kong H, Xiao Q, Yang M, Zu W (2020). Awareness on knowledge about coronavirus disease 2019 prevention and control among residents in Chaoyang district of Beijing City. Article in Chinese. Chin J Public Health.

[49] Fung IC, Cairncross S (2006). Effectiveness of handwashing in preventing SARS: a review. Trop Med Int Health.

[50] De Wandel D, Maes L, Labeau S, Vereecken C, Blot S (2010). Behavioral determinants of hand hygiene compliance in intensive care units. Am J Crit Care.

[51] Lau JTF, Yang X, Tsui HY, Kim JH (2005). Impacts of SARS on health-seeking behaviors in general population in Hong Kong. Prev Med.

[52] Curtis VA, Danquah LO, Aunger RV (2009). Planned, motivated and habitual hygiene behaviour: an eleven country review. Health Educ Res.

[53] Webb TL, Sheeran P (2006). Does changing behavioral intentions engender behavior change? A meta-analysis of the experimental evidence. Psychol Bull.

[54] Jones JH, Salathé Marcel (2009). Early assessment of anxiety and behavioral response to novel swine-origin influenza A(H1N1). PLoS One.

[55] Barr M, Raphael B, Taylor M, Stevens G, Jorm L, Giffin M, Lujic S (2008). Pandemic influenza in Australia: using telephone surveys to measure perceptions of threat and willingness to comply. BMC Infect Dis.

[56] Eastwood K, Durrheim D, Francis JL, d'Espaignet ET, Duncan S, Islam F, Speare R (2009). Knowledge about pandemic influenza and compliance with containment measures among Australians. Bull World Health Organ.

[57] Tang CS, Wong C (2004). Factors influencing the wearing of facemasks to prevent the severe acute respiratory syndrome among adult Chinese in Hong Kong. Prev Med.

[58] Lau JTF, Kim JH, Tsui HY, Griffiths S (2007). Anticipated and current preventive behaviors in response to an anticipated human-to-human H5N1 epidemic in the Hong Kong Chinese general population. BMC Infect Dis.

[59] Hsu HY, Gallinagh R (2001). The relationships between health beliefs and utilization of free health examinations in older people living in a community setting in Taiwan. J Adv Nurs.

[60] Lagerlund M, Hedin A, Sparén P, Thurfjell E, Lambe M (2000). Attitudes, beliefs, and knowledge as predictors of nonattendance in a Swedish population-based mammography screening program. Prev Med.

[61] Nie S, Cao J, Tuo A, Zheng Y (2020). Public's status of KAP for COVID-19 and its influencing factors. Article in Chinese. Shanghai Journal of Preventive Medicine.

[62] Bronfenbrenner U (1977). Toward an experimental ecology of human development. Am Psychol.

[63] Wenzel RP, Edmond MB (2003). Managing SARS amidst uncertainty. N Engl J Med.

[64] Berkman LF, Glass T, Brissette I, Seeman TE (2000). From social integration to health: Durkheim in the new millennium. Soc Sci Med.

[65] Leung GM, Quah S, Ho L, Ho S, Hedley AJ, Lee H, Lam T (2004). A tale of two cities: community psychobehavioral surveillance and related impact on outbreak control in Hong Kong and Singapore during the severe acute respiratory syndrome epidemic. Infect Control Hosp Epidemiol.

[66] Bandura A (1977). Self-efficacy: toward a unifying theory of behavioral change. Psychol Rev.

[67] Lau JTF, Griffiths S, Au DWH, Choi KC (2011). Changes in knowledge, perceptions, preventive behaviours and psychological responses in the pre-community outbreak phase of the H1N1 epidemic. Epidemiol Infect.

[68] Wise T, Zbozinek T, Michelini G, Hagan C Changes in risk perception and protective behavior during the first week of the COVID-19 pandemic in the United States. PsyArXiv.

[69] Jernigan JC, Trauth JM, Neal-Ferguson D, Cartier-Ulrich C (2001). Factors that influence cancer screening in older African American men and women: focus group findings. Fam Community Health.

[70] Wardle J, Williamson S, McCaffery K, Sutton S, Taylor T, Edwards R, Atkin W (2003). Increasing attendance at colorectal cancer screening: testing the efficacy of a mailed, psychoeducational intervention in a community sample of older adults. Health Psychol.

[71] Booth M, Bauman A, Owen N (2020). Perceived barriers to physical activity among older Australians. J Aging Phys Act.

[72] Lau JTF, Yang X, Tsui HY, Pang E (2004). SARS related preventive and risk behaviours practised by Hong Kong-mainland China cross border travellers during the outbreak of the SARS epidemic in Hong Kong. J Epidemiol Community Health.

[73] Lau JTF, Yang X, Tsui H, Kim JH (2003). Monitoring community responses to the SARS epidemic in Hong Kong: from day 10 to day 62. J Epidemiol Community Health.

[74] Smith Klohn L, Rogers RW (1991). Dimensions of the severity of a health threat: the persuasive effects of visibility, time of onset, and rate of onset on young women's intentions to prevent osteoporosis. Health Psychol.

[75] Aoun S, Donovan RJ, Johnson L, Egger G (2002). Preventive Care in the Context of Men's Health. J Health Psychol.

[76] Ronis DL (1992). Conditional health threats: health beliefs, decisions, and behaviors among adults. Health Psychol.

